# Impact of hall and ion slip in a thermally stratified nanofluid flow comprising Cu and Al_2_O_3_ nanoparticles with nonuniform source/sink

**DOI:** 10.1038/s41598-020-74510-1

**Published:** 2020-10-22

**Authors:** Nosheen Gul, Muhammad Ramzan, Jae Dong Chung, Seifedine Kadry, Yu-Ming Chu

**Affiliations:** 1 Department of Computer Science, Bahria University, Islamabad, 44000 Pakistan; 2 Department of Mechanical Engineering, Sejong University, Seoul, 143-747 Korea; 3 Department of Mathematics and Computer Science, Faculty of Science, Beirut Arab University, 115020 Beirut, Lebanon; 4 Department of Mathematics, Huzhou University, Huzhou, 313000 People’s Republic of China; 5 Hunan Provincial Key Laboratory of Mathematical Modeling and Analysis in Engineering, Changsha University of Science and Technology, Changsha, 410114 People’s Republic of China

**Keywords:** Mathematics and computing, Engineering, Mechanical engineering

## Abstract

Nanofluids play a pivotal role in the heat transport phenomenon and are essential in the cooling process of small gadgets like computer microchips and other related applications in microfluidics. Having such amazing applications of nanofluids, we intend to present a theoretical analysis of the thermally stratified 3D flow of nanofluid containing nano solid particles (Cu and Al_2_O_3_) over a nonlinear stretchable sheet with Ion and Hall slip effects. Moreover, the features of buoyance effect and non-uniform heat source/skin are also analyzed. For the study of numerically better results, Tawari and Das model is adopted here. For the conversion of the system of partial differential equations into ordinary differential equations, apposite transformations are engaged and are tackled by utilizing the bvp4c scheme of MATLAB software. The effects of dimensionless parameters on velocity and temperature profiles are depicted with the help of graphs. Additionally, the Skin friction coefficient and Nusselt number for the practical applications are examined in the tabular form. Verification of the current study by comparing it with an already published work in a special case is also a part of this study. Results show that the thermal performance of copper nanoparticles is more than alumina nanoparticles. An upsurge in the temperature of nanofluid is observed when the strength of the magnetic field is enhanced. However, the temperature of partially ionized nanofluid is significantly lowered because of the collisions of electrons and ions.

## Introduction

The vitality of heat energy transportation is well recognized in manufacturing industrial processes. The poor rate of heat transportation can affect the efficiency of the thermal cooling system. Conventional liquids like water, ethylene glycol, and oil due to lower thermal conductivity, pose limitations in efficiency and compactness of a variety of engineering equipment like electronic devices and heat exchangers. These limitations have instigated the development of advanced fluids with higher conductivity and heat transfer capacity. Thermal conductivities of liquids are enhanced by an advanced method of suspending tiny solid particles in the fluid i.e., adding metallic, non-metallic, and polymeric particles into the customary fluids. These liquids with suspended particles are predicted to have higher thermal conductivity to that of normal liquids. Nanofluids are modernized type of fluids with stable and homogenous dispersion of a certain amount of nano-sized (< 100 nm) particles. The suspension of solid nanoparticles even in smaller amounts in conventional liquids amazingly enhances their thermal conductivity. Increasing the rate of heat transfer in a variety of cases, the nanofluids depict great potential in comparison to the usual techniques available for enhancement of heat transfer^1^. Daily life applications influenced by nanofluids may include the cooling of atomic reactors and transformers. In medical, the usage of magneto nanofluids like in hyperthermia, magnetic resonance imaging, and in the treatment of cancer is also well recognized. Moreover, in the removal of tumors, and the manufacturing of germs free surgical instruments, nanofluid in the fundamental gradient. It is evident from the literature that the two-phase or single-phase approach is adopted for modeling of heat transfer with nanofluids. According to the two-phase approach, velocity might not be zero between fluid and particles^2^ owing to various factors like Brownian forces, friction among fluid and particles, Brownian diffusion, sedimentation, dispersion, and gravity. Nanoparticles, in a single-phase approach, can easily be fluidized for this reason it may be assumed that motion slip, if any, would be considered negligible between two phases^3^. The latter scheme is computationally simpler and more efficient. Nayak et al.^4^ studied the thermal conductivity of 3D nanofluid flow by using a single-phase model namely the Patel model with the effect of buoyance, transverse magnetic field, and thermal radiation over a linearly stretching sheet. Patel model is implemented on the electrically conducting nanofluid flow over an exponentially stretching sheet with the impact of the variable magnetic field by Nayak et al.^5^. Tawari and Das single-phase model is well known for computational research and for the characterization of nanoparticle volume fraction to elaborate the flow and heat transit processes^6^. Several recent explorations underline the importance of nanofluids. Usman et al.^7^ scrutinized the significant effects of nonlinear thermal radiation and bouncy forces on the flow of hybrid nanofluid past a 3D stretching sheet. Reddy et al.^8^ reckoned the numerical outcome of 3D flow with impacts of non-uniform heat source/sink and Joule heating on non-Newtonian nanofluid past a linearly stretchable geometry. Models depicting the hybrid nanofluid fluid flow comprising Cu and Al_2_O_3_ nanoparticles with varied geometries may be found in^7,9–14^. Further, Rasool et al.^15^ reported the MHD Darcy flow of Williamson nanofluid with entropy generation and binary chemical reaction along with Arrhenius activation energy. Several studies of nanofluids can be found in these references^16–21^. Khan et al.^22^ considered the impact of Darcy Forchheimer on three-dimensional fluid flow with the amalgamation of carbon nanotubes over an exponentially stretched surface. Researchers have adopted this model in several explorations^23–30^.

Completely different characteristics are exhibited by the partially ionized liquid to that of natural fluids when exposed to the magnetic field. A partially ionized fluid influenced by the magnetic field experiences three types of forces namely the Hall force owing to electrons’ collision, magnetic force, and the Ion force because of ion collision. It is also verified experimentally that Hall and Ion’s forces act in an opposite direction to that of a magnetic force. These Hall current and Ion slip forces may be found in a partially ionized fluid by employing the Ohm law^31^ along with other basic laws with the Maxwell equations. Moreover, some studies on the magnetohydrodynamic flow of partially ionized liquid adopting stated laws^32^ have been discussed. Nasrin et al.^33^ studied the incompressible 2D partially ionized fluid flow in the existence of a transverse magnetic field along with a rotating semi-infinite vertical plate with a porous medium by utilizing the perturbation scheme. Takhar et al.^34^ studied the Hall current impact under the influence of the magnetic field amalgamated with free stream velocity in a non-similar flow past a moving surface. Opanuga et al.^35^ analyzed the irreversibility of incompressible couple stress fluid flow along a microchannel with porous medium with the impact of Hall and Ion slip current by utilizing analytical method (Differential transform method). Recently some studies of partially ionized fluid were conducted on a three-dimensional stretching surface which is highlighted below. Nawaz et al.^36^ analyzed the 3D thermal performance and simulating numerical results through Galerkin Finite Element Method (GFEM). The unsteady MHD free convective rotating flow over an exponentially accelerated plate with effects of Hall and ion slip through a saturated porous medium is investigated by Krishna et al.^37^. Nawaz et al.^38^ by using FEM (Finite Element Method) in their investigation, draw a comparison of Carreau nanofluid and hybrid nanofluid flow by adopting Tawari and Das mathematical model with the effect of Hall and Ion current. The numerical solution of the flow of magnetized partially ionized fluid with an amalgamation of nano-sized particles considering the effects of non-uniform heat source, thermal radiations, and heterogeneous homogeneous reactions, is investigated by Nawaz et al.^39^. Some recent studies highlighting the impacts of Hall current may be found in^40–44^.

The process of thermal stratification originates because of temperature variation which results in affecting the density of the medium. The fluids bodies which are surrounded by heated walls having variant temperatures possess the physical properties of thermal stratification and this phenomenon has drawn the attention of researchers for the last few decades. Flows with thermal stratification have important applications in the fields of industries, agriculture fields, oceanography, volcanic flows, geo-thermal systems, lake thermo-hydraulics, etc. Tlili et al.^45^ investigated the dual stratified flow with Maxwell nanofluid past a bidirectional stretched sheet under the effects of heat source/sink and chemical reactions by using an analytic technique (Homotopy Analysis Method). Hayat et al.^46^ examined the thermally stratified 3D MHD flow over a stretchable exponential sheet with the impact of Joule heating and viscous dissipation by utilizing HAM. Ramzan et al.^47^ analyzed the dual stratified flow of micropolar nanofluid through a vertically stretched sheet. They also carried out buoyance effect and thermal radiation for flow analysis by using the Runge–Kutta technique in Maple software. Alshomrani et al.^48^ studied the stratified Bio-convective nanofluid flow with the dispersion of carbon nanotube over a stretchable cylinder under the influence of ferromagnetic dipole. The impact of slip on the three-dimensional flow of Williamson nanofluid over a linear stretched surface is analytically analyzed by Ramzan et al.^49^. The flow problem is strengthened by carrying out double stratification and Cattaneo–Christov heat flux. Furthermore, some studies elaborating on stratified nanofluid are discussed through^50–54^.

Theoretical study of fluid flows influenced by magnetohydrodynamic (MHD) has been a subject of potential interest owing to its wide-ranging applications in the designing of MHD generators, cooling system with liquid metals, pumps, flow meters, and accelerators, etc. Seyedi et al.^55^ found a numerical solution of the Eyring-Powell fluid flow with chemical reaction and magnetohydrodynamics in a stretching channel using Galerkin based multiscale scheme. The viscous fluid flow of over a bi-directional stretched surface owing to an impulsive motion of a stretched surface is studied by Takhar et al.^56^. It is comprehended here that the magnetic field effects are more dominated in the *y-*direction on the surface. Further, Magyari and Chamkha^57^ analyzed the Marangoni convective flow with thermal and solutal stratifications at the boundary of the stretched surface. The time dependent flow and heat transfer with an aligned magnetic field on a semi-infinite flat plate is examined by Takhar et al.^58^. Chamkha and Khaled^59^ studied the hydromagnetic mixed convection Hiemenz flow in a permeable medium.

Given the foregoing, it is pertinent to mention that abundant studies may be quoted featuring the nanofluid 2D flows with immersed simple/hybrid nanoparticles over linear stretched surfaces. And few are available when we talk about 3D hybrid nanofluid flows. But no study so far is performed that discusses the 3D hybrid nanofluid flow with Hall current and Ion slip over a nonlinear stretched surface influenced by nonuniform source/sink in thermally stratified media. A comparison Table 1 is given to reflect the exact novelty of the presented model with the existing available literature. So, in this exploration, we analyzed the thermally stratified 3D partially ionized nanofluid by containing nano solid particle (Cu and Al_2_O_3_) past a nonlinear stretchable sheet. We make the current study unique by considering the buoyance effect and non-uniform heat source/skin. The envisioned model is supported by the thermal stratification condition at the boundary of the extended surface. The governing equations are numerically solved after similarity transformation by utilizing the bvp4c MATLAB package. The impacts on velocity and temperature profile by the dimensionless governing parameters are debated and presented with the help of graphs. Moreover, the coefficient of Skin friction and Nusselt number are examined and shown by Table.

**Table 1:** Literature survey for uniqueness of the presented model.

Authors	3D model	Cu/Al_2_O_3-_water	Hall/Ion slip effect
Usman et al.^7^	√	√	×
Devi and Devi^9^	×	√	×
Devi and Devi^10^	√	√	×
Ghadikolaei et al.^11^	×	√	×
Venkateswarlu and Narayana^12^	×	√	×
Prakash and Devi^13^	×	√	×
Lund et al.^14^	×	√	×
Present	√	√	√

## Mathematical formulation

**Figure 1:**
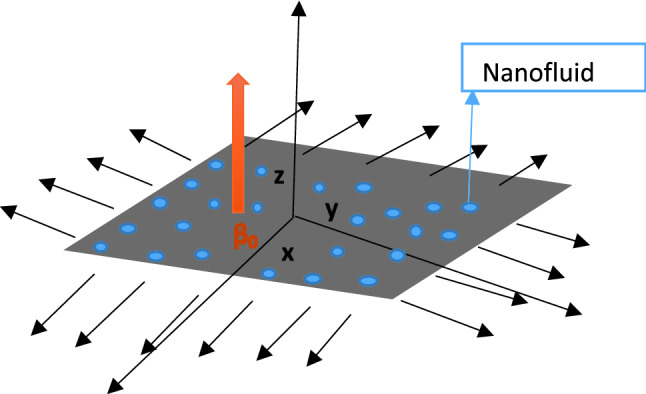
Physical diagram with the coordinate system.

We study the flow of magnetized nanofluid with nano solid particles of Cu (Copper) and Al_2_O_3_ (Aluminum oxide) over a three-dimensional nonlinear stretchable sheet. The Buoyance effect and nonlinear heat source/sink with thermal stratification on boundary conditions are also considered. The stretching velocity $V_{w}$ = $[a({\text{y}} + x)^{m} , \, b({\text{y}} + x)^{m} ]$ and temperature at the wall and ambient $T_{w} = T_{0} + a_{1} x,$ $T_{\infty } = T_{0} + b_{1} x$ are given with constants ($a,$ $b,$ $m,$ $a_{1}$ and $b_{1}$). A present magnetic field $B_{0} = [0, \, 0, \, \beta_{0} ]$ is carried out along the *z-*axis. A significant role of Joule heating and viscous dissipation is assumed. The electric and magnetic fields are ignored and the Reynolds number to be considered very small. The conversation equations of mass, momentum, and energy under the assumption are given as:

1 $$ \nabla \cdot U = 0, $$

2 $$ \rho_{nf} \frac{{{{\text{d}\overline{\text{U}}}}}}{{{\text{dt}}}} = - \nabla p + \overline{J} \times B_{0} + \mu_{nf} \nabla^{2} \overline{U} + g\beta_{nf} (\overline{\text{T}} - \overline{\text{T}}_{\infty } ), $$

3 $$ \left( {\rho C_{p} } \right)_{nf} \frac{{d\overline{T}}}{dt} = k_{nf} \nabla^{2} \overline{T} + \frac{1}{{\sigma_{nf} }}\overline{J} \cdot \overline{J} + tr(\tau \overline{L}) + Q^{\prime \prime \prime } $$

4 $$ \frac{{\partial B_{0} }}{\partial t} = \nabla \times \overline{E}, \, \mu_{1} \overline{J} = \nabla \times B_{0} , \, \nabla \cdot B_{0} = 0, $$

5 $$ \overline{J} = \frac{{\beta_{H} \beta_{i} }}{{\left| {B_{0} } \right|^{2} }}(\overline{J} \times B_{0} ) \times B_{0} - \frac{{\beta_{H} }}{{\left| {B_{0} } \right|}}(\overline{\text{J}} \times B_{0} ) + \sigma_{nf} \left[ {\overline{E} + \overline{U} \times B_{0} } \right], $$

where the non-uniform heat abortion/generation parameter, magnetic field, Hall parameter, velocity gradient, ion slip parameter, pressure and velocity, current density, temperature, thermal expansion, thermal conductivity, electrical conductivity, dynamic viscosity, and specific heat of the nanofluid are symbolized as $Q^{\prime \prime \prime }$, $B_{0}$, $\beta_{H}$, $L$, $\beta_{i}$, $p$, $\overline{U}$, $\overline{J}$, $\overline{T}$, $\beta_{nf}$, $k_{nf}$, $\sigma_{nf}$, $\mu_{nf}$ and $(\rho C_{p} )_{nf}$ respectively. The schematic geometry of the problem is demonstrated in Fig. 1.

The bi-directional flow under boundary layer equations are given by:

6 $$ U_{x} + V_{y} + W_{z} = 0, $$

7 $$ UU_{x} + VU_{y} + WU_{z} = \upsilon_{nf} U_{zz} + g\frac{{(\rho \beta )_{nf} }}{{\rho_{nf} }}({\text{T}} - {\text{T}}_{\infty } ) + \frac{{\sigma_{nf} \beta_{0}^{2} ({\text{y}} + x)^{m - 1} [{\text{V}}\beta_{e} - {\text{U}}(1 + \beta_{i} \beta_{e} )]}}{{\rho_{nf} [(1 + \beta_{e} \beta_{i} )^{2} + (\beta_{e} )^{2} ]}}, $$

8 $$ UV_{x} + VV_{y} + WV_{z} = \upsilon_{nf} V_{zz} + g\frac{{(\rho \beta )_{nf} }}{{\rho_{nf} }}({\text{T}} - {\text{T}}_{\infty } ) - \frac{{\sigma_{nf} \beta_{0}^{2} ({\text{y}} + {{x}})^{m - 1} [{\text{U}}\beta_{e} + V(1 + \beta_{i} \beta_{e} )]}}{{\rho_{nf} [(1 + \beta_{e} \beta_{i} )^{2} + (\beta_{e} )^{2} ]}}, $$

9 $$ UT_{x} + VT_{y} + WT_{z} = \frac{{K_{nf} }}{{(\rho C_{p} )_{nf} }}T_{zz} + \frac{{\mu_{nf} }}{{(\rho C_{p} )_{nf} }}[{\text{U}}_{z}^{2} + {\text{V}}_{z}^{2} ] + \frac{{\sigma_{nf} \beta_{0}^{2} (y + x)^{m - 1} [{\text{V}}^{2} + {\text{U}}^{2} ]}}{{(\rho C_{p} )_{nf} [(1 + \beta_{e} \beta_{i} )^{2} + (\beta_{e} )^{2} ]}} + \frac{{Q^{*} }}{{(\rho C_{p} )_{nf} }}. $$

No-slip condition and thermal stratification are taken as boundary conditions of the above proposed mathematical model.

10 $$ \begin{aligned} & \left. U \right|_{z = 0} = U_{w} = a(y + x)^{m} , \, \left. V \right|_{z = 0} = V_{w} = b(y + x)^{m} , \, \left. W \right|_{z = 0} { = 0 }\left. T \right|_{z = 0} = T_{w} = T_{0} + a_{1} (y + x)^{m} \\ & \left. U \right|_{z \to \infty } = 0, \, \left. T \right|_{z \to \infty } = T_{\infty } = T_{0} + b_{1} (y + x)^{m} . \\ \end{aligned} $$

Note that components of fluid velocities along *x, y,* and *z* directions, fluid temperature, and magnetic field are denoted by $(U, \, V \, \& \, W)$, $T$ and $\beta_{0}$. The non-uniform heat abortion/generation parameter $Q^{*}$ is given below:

11 $$ Q^{*} = \left[ {\frac{{k_{f} {\text{U}}_{w} }}{{\upsilon_{f} (y + x)^{m} }}\left\{ {{\text{A}}_{1} ({\text{T}} - {\text{T}}_{\infty } ) + {\text{B}}_{2} ({\text{T}}_{W} -{\text{ T}}_{\infty } )} \right\}} \right], $$

where temperature and space heat generation/absorption parameters are A_1_ and B_2_, respectively. Similarity transformations are defined by:

12 $$ \begin{aligned} & U = a(y + x)^{m} F^{\prime } ,\quad {\text{V } = \text{ }}a(y + x)^{m} G^{\prime } ,\quad W = - \sqrt {a\upsilon_{f} (y + x)^{m} } \left[ {\frac{m + 1}{2}({\text{F}} + {\text{G}}) + \frac{m - 1}{2}\xi ({\text{F}}^{\prime } + G^{\prime } )} \right], \\ & T = T_{\infty } + (T_{w} - T_{0} )\Theta ,\quad \xi = - \sqrt {\frac{a}{{\upsilon_{f} }}(y + x)^{m} } z. \\ \end{aligned} $$

Table 2 is displayed to show the relationship between the physical traits of the nanoparticles, nano-liquids, and base fluids that can be described by various models. The following model has been adopted in the current study by^53^.

**Table 2:** The model for thermophysical features^7,9–14^.

Properties	Base fluid and solid nanoparticle
Viscosity	$\mu_{nf} = \mu_{f} (1 - \chi )^{ - 2.5}$
Heat capacity	$\frac{{(\rho C_{p} )_{nf} }}{{(\rho C_{p} )_{f} }} = (1 - \chi ) + \chi \frac{{(\rho C_{p} )_{s} }}{{(\rho C_{p} )_{f} }},$
Density	$\frac{{\rho_{nf} }}{{\rho_{f} }} = (1 - \chi ) + \chi \frac{{\rho_{s} }}{{\rho_{f} }},$
Thermal conductivity	$\frac{{k_{nf} }}{{k_{f} }} = \frac{{(k_{s} + 2k_{f} ) + 2\chi (k_{s} \, - k_{f} )}}{{(k_{s} + 2k_{f} ) - \chi (k_{s} \, - k_{f} )}},$
Electrical conductivity	$\frac{{\sigma_{nf} }}{{\sigma_{f} }} = \left( {1 + \frac{{3\left( {\sigma_{s} - \sigma_{f} } \right)\chi }}{{\sigma_{s} + 2\sigma_{f} + \left( {\sigma_{f} - \sigma_{s} } \right)\chi }}} \right)$
Thermal expansion coefficient	$\frac{{(\rho \beta )_{nf} }}{{(\rho \beta )_{f} }} = (1 - \chi ) + \chi \frac{{(\rho \beta )_{s} }}{{(\rho \beta )_{f} }},$

After applying similarity transformation on governing equations, continuity Eq. (6) is satisfied, and Eqs. (7)–(9) take the form:

13 $$ F^{{{\prime \prime \prime }}} + \phi_{1} \left[ {\left( {\frac{m + 1}{2}} \right)F^{{{\prime \prime }}} ({\text{F}} + {\text{G}}) - mF^{{\prime }} ({\text{F}}^{{\prime }} + {\text{G}}^{{\prime }} )} \right] + \phi_{2} M^{2} \frac{{\left[ {\beta_{e} G^{{\prime }} - \left( {1 + \beta_{e} \beta_{i} } \right)F^{{\prime }} } \right]}}{{\left[ {\left( {1 + \beta_{e} \beta_{i} } \right)^{2} + \left( {\beta_{e} } \right)^{2} } \right]}} + \phi_{3} R_{i} \Theta = 0, $$

14 $$ {\text{G}}^{{{\prime \prime \prime }}} + \phi_{1} \left[ {\left( {\frac{m + 1}{2}} \right){\text{G}}^{{{\prime \prime }}} ({\text{F}} + {\text{G}}) - mG^{{\prime }} ({\text{F}}^{{\prime }} + {\text{G}}^{{\prime }} )} \right] - \phi_{2} M^{2} \frac{{\left[ {\beta_{e} {\text{F}}^{{\prime }} + \left( {1 + \beta_{e} \beta_{i} } \right){\text{G}}^{{\prime }} } \right]}}{{\left[ {\left( {1 + \beta_{e} \beta_{i} } \right)^{2} + \left( {\beta_{e} } \right)^{2} } \right]}} + \phi_{3} R_{i} \Theta = 0, $$

15 $$ \begin{aligned} & \frac{{k_{nf} \mu_{f} }}{{k_{f} \mu_{nf} \Pr }}\left[ {\Theta^{\prime \prime } + A_{1} \Theta + B_{2} \left( {1 - S_{t} } \right)F^{\prime}} \right] + \phi_{4} \left[ {\left( {\frac{m + 1}{2}} \right)\Theta^{\prime } ({\text{F}} + {\text{G}}) - m(1 + {\text{S}}_{t} )\Theta ([{\text{F}}^{\prime } + {\text{G}}^{\prime } )} \right] \\ & \quad + \phi_{2} M^{2} Ec\frac{{\left[ {({\text{F}}^{\prime } )^{2} + ({\text{G}}^{\prime } )^{2} } \right]}}{{\left[ {\left( {1 + \beta_{e} \beta_{i} } \right)^{2} + \left( {\beta_{e} } \right)^{2} } \right]}} + Ec\left[ {\left( {F^{{\prime {\prime }}} } \right)^{2} + \left( {G^{{\prime {\prime }}} } \right)^{2} } \right] = 0, \\ \end{aligned} $$

with associated boundary conditions

16 $$ F(0) = G(0) = 0, \, F^{\prime } (0) = 1, \, G^{\prime } (0) = \lambda , \, \Theta = {1} - S_{t} ,\quad F^{\prime } (\infty ) = {\text{G}}^{\prime } (\infty ) = \Theta (\infty ) = 0. $$

When $\lambda = 0$ the mentioned equation is changed to describe the 2D flow. The differential system which leads the flow of a nanofluid in the symmetric axis caused by a nonlinearly stretching surface is improved when $\lambda = 1$.

Here, the volumetric expansion rates, Richardson Number, Magnetic parameter, Eckert number, thermal stratification, Grashof number, Prandtl number, stretching ratio parameter, and Reynolds number are defined as under:

17 $$ \begin{aligned} & \phi_{1} = (1 - \chi )^{2.5} \left( {1 - \chi + \chi \frac{{\left( \rho \right)_{s} }}{{\left( \rho \right)_{f} }}} \right),\quad \phi_{3} = (1 - \chi )^{2.5} \left( {1 - \chi + \chi \frac{{\left( {\rho \beta } \right)_{s} }}{{\left( {\rho \beta } \right)_{f} }}} \right),\quad \phi_{5} = (1 - \chi )^{ - 2.5} , \\ & \phi_{2} = (1 - \chi )^{2.5} \left( {1 + \frac{{3(\sigma_{s} - \sigma_{f} )\chi }}{{\sigma_{s} + 2\sigma_{f} + (\sigma_{f} - \sigma_{s} )\chi }}} \right),\quad \phi_{4} = (1 - \chi )^{2.5} \left( {1 - \chi + \chi \frac{{\left( {\rho C_{p} } \right)_{s} }}{{\left( {\rho C_{p} } \right)_{f} }}} \right), \\ & R_{i} = \frac{{G_{r} }}{{{\text{Re}}_{x}^{2} }},\quad M = \sqrt {\frac{{\sigma_{f} \beta_{0}^{2} }}{{e\rho_{f} }}} ,\quad E_{c} = \left( {\frac{{U_{w}^{2} }}{{C_{p} (T_{w} - T_{0} )}}} \right),\quad S_{t} = \frac{{b_{1} }}{{a_{1} }},\quad G_{r} = \frac{{g(\rho \beta )_{f} (T - T_{\infty } )U_{w}^{\frac{3}{m}} }}{{a^{\frac{3}{m}} \upsilon_{f}^{2} }},\quad Pr = \frac{{\mu_{f} ({\text{C}}_{p} )_{f} }}{{k_{f} }},\quad Re = \frac{{U_{w}^{{^{{(1 + \frac{1}{m})}} }} }}{{a^{\frac{1}{m}} \upsilon_{f} }}. \\ \end{aligned} $$

Table 3 is erected to portray various properties of the base fluid and nano solid particles have been adopted in the current study by Sheikholeslami and Ganji^60^.

**Table 3:** Values of the base fluid, and nanoparticles for thermophysical characteristics Sheikholeslami and Ganji^60^.

Thermophysical properties	Pure water	Alumina (Al_2_O_3_)	Copper (Cu)
$\rho \,({\text{kg}}\,{\text{m}}^{ - 3} )$	997.1	3970	8933
$\beta \times 10^{5} \,(K^{ - 1} )$	21	0.85	1.67
$k\,(Wm^{ - 1} K^{ - 1} )$	0.613	40	401
$\sigma \,(\Omega \,{\text{m}})^{ - 1}$	0.05	1 × 10^–10^	5.96 × 10^7^
${\text{C}}_{p} \,(J\,\,kg^{ - 1} \,K^{ - 1} )$	4179	765	385

For the practical application, we take the physical quantities like Skin friction coefficient and Nusselt number:

18 $$ C_{Fx} = \frac{{\tau_{xz} }}{{\rho_{f} U_{{_{w} }}^{2} }}, \, C_{Gy} = \frac{{\tau_{yz} }}{{\rho_{f} V_{{_{w} }}^{2} }}, \, Nu = \frac{{(y + x)q_{w} }}{{(T_{w} - T_{\infty } )}}, $$

The shear stresses of the wall along *x, y-*directions, and heat flux are as follow respectively:

19 $$ \tau_{xz} = \mu_{nf} \left. {(U_{z} + W_{x} )} \right|_{z = 0} , \, \tau_{yz} = \mu_{nf} \left. {(U_{z} + W_{y} )} \right|_{z = 0} , \, q_{w} = - k_{nf} \left. {\left( {T_{z} } \right)} \right|_{z = 0} , $$

The dimensionless form of Eq. (18) by applying Eq. (12) becomes as:

20 $$ (Re)^{0.5} C_{{F_{x} }} = \phi_{5} F^{{{\prime \prime }}} (0), \, (Re)^{0.5} C_{{G_{y} }} = \phi_{5} G^{{{\prime \prime }}} (0),{\text{ (Re}})^{0.5} Nu = - \frac{{k_{nf} }}{{k_{f} }}\left( {\frac{1}{{1 - S_{t} }}} \right)\Theta^{{\prime }} (0) $$

## Numerical solution

The highly nonlinear system of coupled differential equations is solved numerically for a better understanding of the envisioned problem. Numerical outcomes of Eqs. (13)–(15) associated with boundary conditions (16) are analyzed by utilizing the MATLAB code namely bvp4c. For this purpose, primarily the Partial differential equations are transformed into ordinary differential equations by adopting new parameters. The system of equations is converted into the first-order system of differential equations associated with the transformed boundary conditions are:

21 $$ U_{1} = F,U_{2} = F^{{\prime }} ,U_{3} = F^{{{\prime \prime }}} ,U_{4} = G,U_{5} = G^{{\prime }} ,U_{6} = G^{{{\prime \prime }}} ,U_{7} = \theta ,U_{8} = \theta^{{\prime }} , $$

22 $$ UU_{1} = - \varphi_{1} \left[ {\left( {\frac{m + 1}{2}} \right)U_{3} (U_{1} + U_{4} ) - mU_{2} (U_{2} + U_{5} )} \right] - \varphi_{2} M^{2} \frac{{[\beta_{e} U_{5} - (1 + \beta_{e} \beta_{i} )U_{2} ]}}{{[(1 + \beta_{e} \beta_{i} )^{2} + (\beta_{e} )^{2} ]}} - \varphi_{3} R_{i} U_{7} , $$

23 $$ UU_{2} = - \varphi_{1} \left[ {\left( {\frac{m + 1}{2}} \right)U_{6} (U_{1} + U_{4} ) - mU_{5} (U_{2} + U_{5} )} \right] + \varphi_{2} M^{2} \frac{{[\beta_{e} U_{2} - (1 + \beta_{e} \beta_{i} )U_{5} ]}}{{[(1 + \beta_{e} \beta_{i} )^{2} + (\beta_{e} )^{2} ]}} - \varphi_{3} R_{i} U_{7} , $$

24 $$ \begin{aligned}  UU_{3} &= \left( { - A_{1} U_{7} - B_{2} (1 - S_{t} } \right)U_{2} - \varphi_{4} \left[ {\left( {\frac{m + 1}{2}} \right)U_{8} \left( {U_{1} + U_{4} } \right) - m(1 + S_{t} )U_{7} (U_{2} + U_{5} )} \right] \\ & \quad - \varphi_{2} M^{2} Ec\frac{{[(U_{2} )^{2} + (U_{5} )^{2} ]}}{{[(1 + \beta_{e} \beta_{i} )^{2} + (\beta_{e} )^{2} ]}} - Ec[(U_{3} )^{2} + (U_{6} )^{2} ], \\ \end{aligned} $$

25 $$ \begin{gathered} U_{0} (1);U_{0} (4);U_{0} (2) - 1;U_{0} (5) - \lambda ;U_{0} (7) - 1 - S_{t} , \hfill \\ U_{\inf } (2);U_{\inf } (5);U_{\inf } (7). \hfill \\ \end{gathered} $$

The bvp4c function necessitates an initial supposition for the explanation and the tolerance for the problem under consideration is taken as $10^{ - 6}$. The selected initial estimate must associate with the boundary condition asymptotically and the solution as well.

The grid independence test of the bvp4c function of the MATLAB software is also performed for the Nusselt number. From Table 4, it can be seen that grid size 200 * 200 is enough for the system to be grid free. After that values of Nusselt number seems to be independent of all grids.

**Table 4:** Grid free analysis for the Nusselt number.

Serial No	Grid size	$Nu_{ave,\theta }$
1	30 × 30	2.40280
2	50 × 50	2.40279
3	100 × 100	2.40278
4	150 × 150	2.40277
5	200 × 200	2.40276
6	300 × 300	2.40276

## Results and discussion

**Figure 2:**
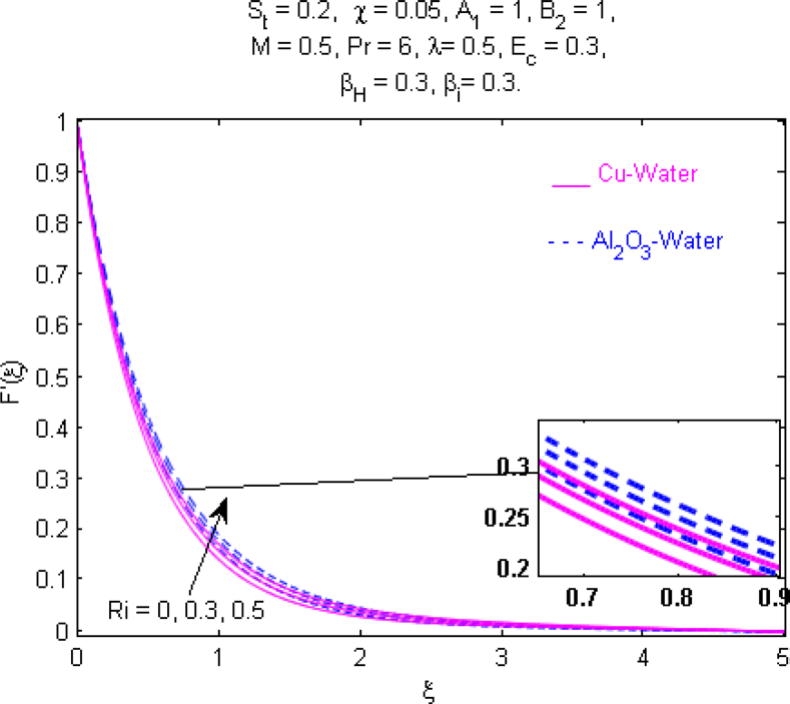
Velocity $F^{\prime } (\xi )$ response for $R_{i}$.

**Figure 3:**
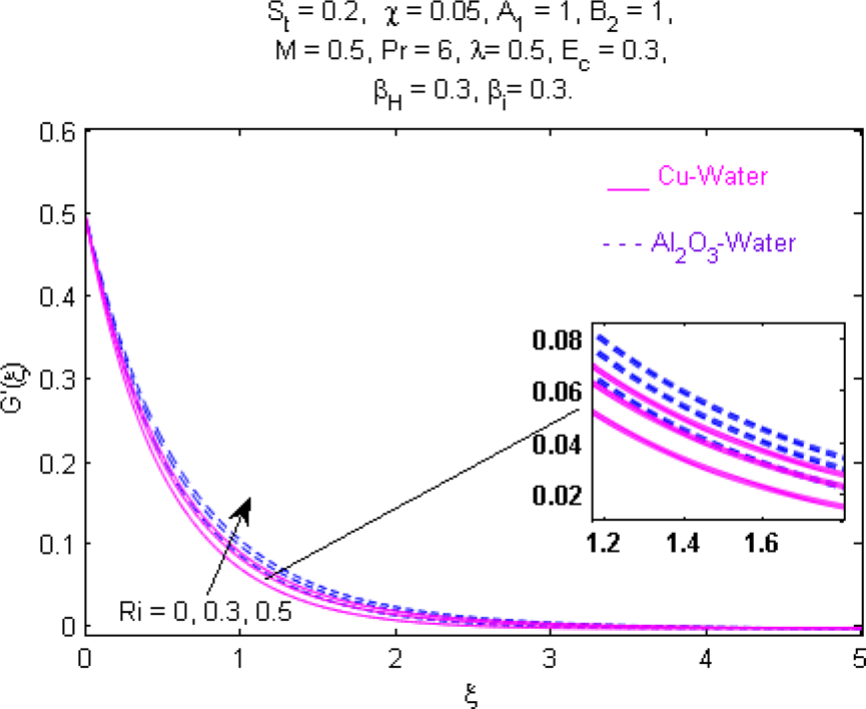
Velocity ${\text{G}}^{\prime } (\xi )$ response for $R_{i}$.

**Figure 4:**
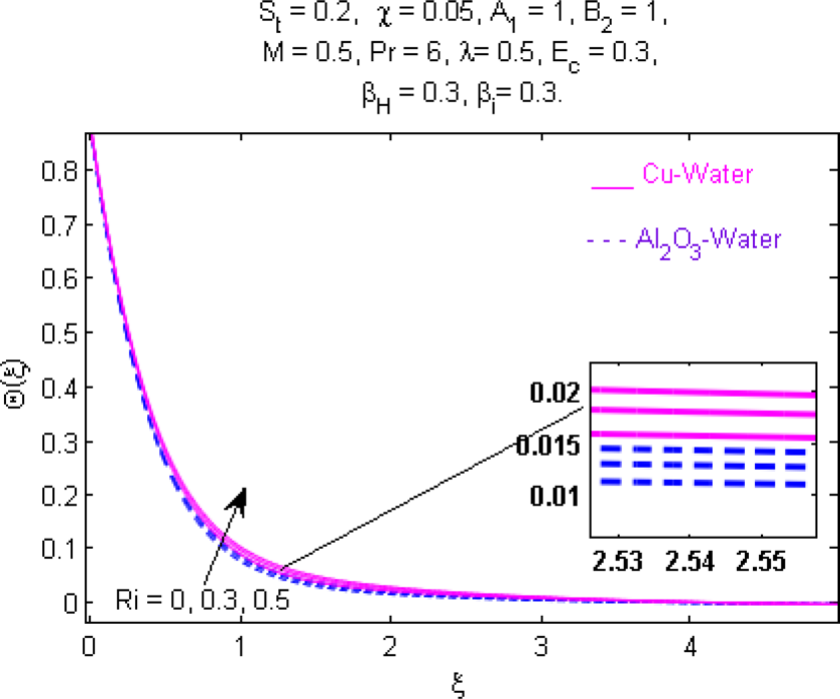
Temperature $\Theta (\xi )$ response for $R_{i}$.

**Figure 5:**
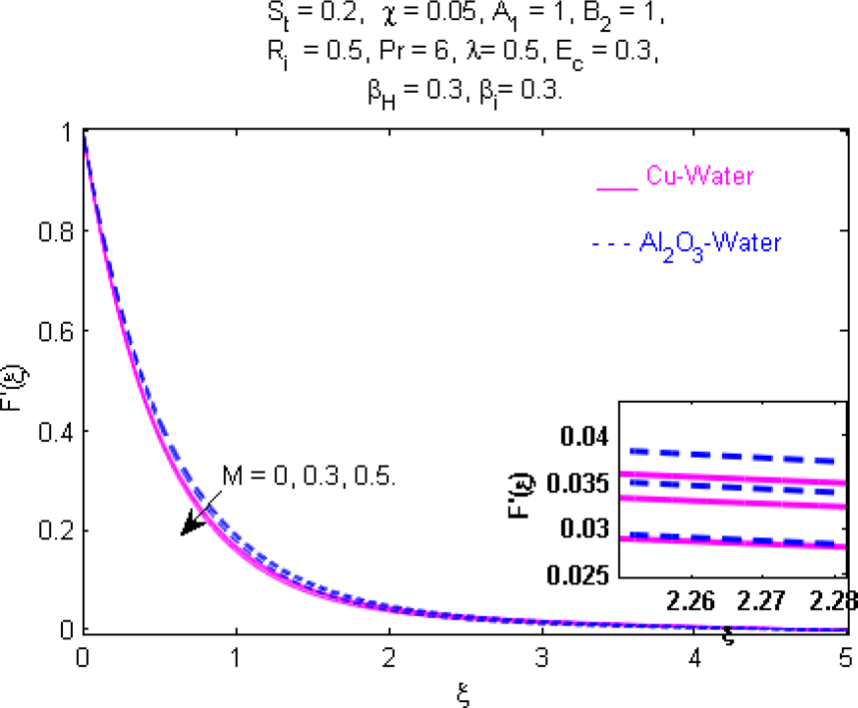
Velocity $F^{\prime } (\xi )$ response for $M$.

**Figure 6:**
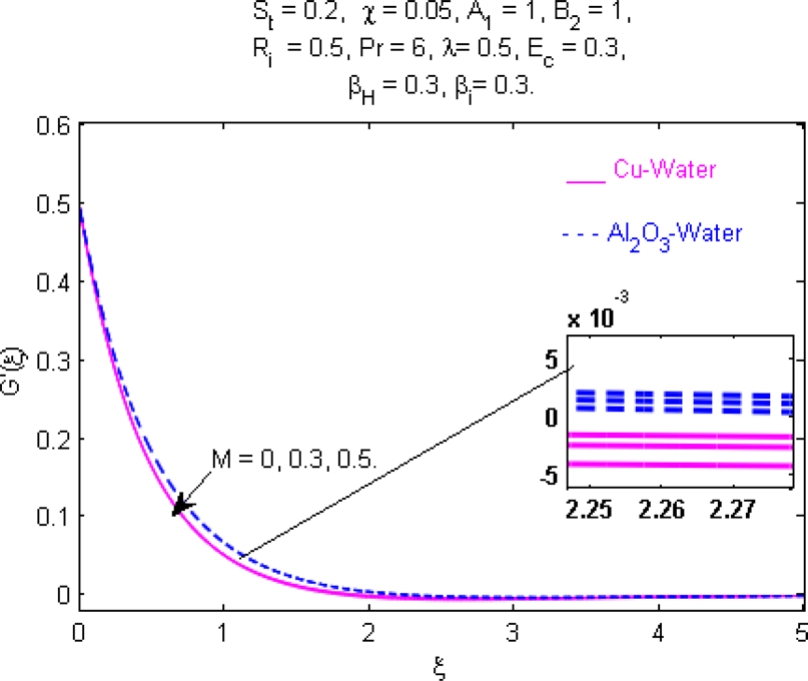
Velocity ${\text{G}}^{\prime } (\xi )$ response for $M$.

**Figure 7:**
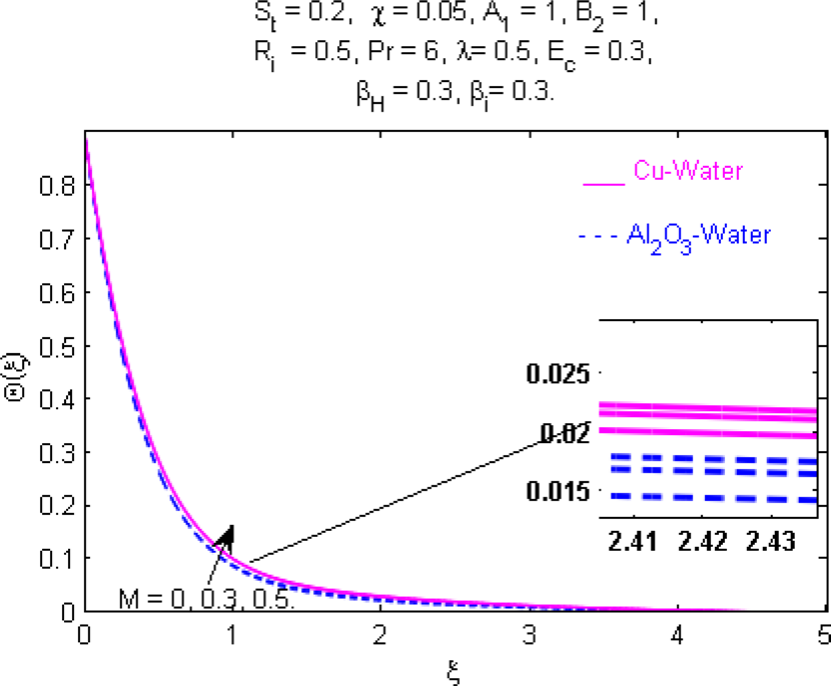
Temperature $\Theta (\xi )$ response for $M$.

**Figure 8:**
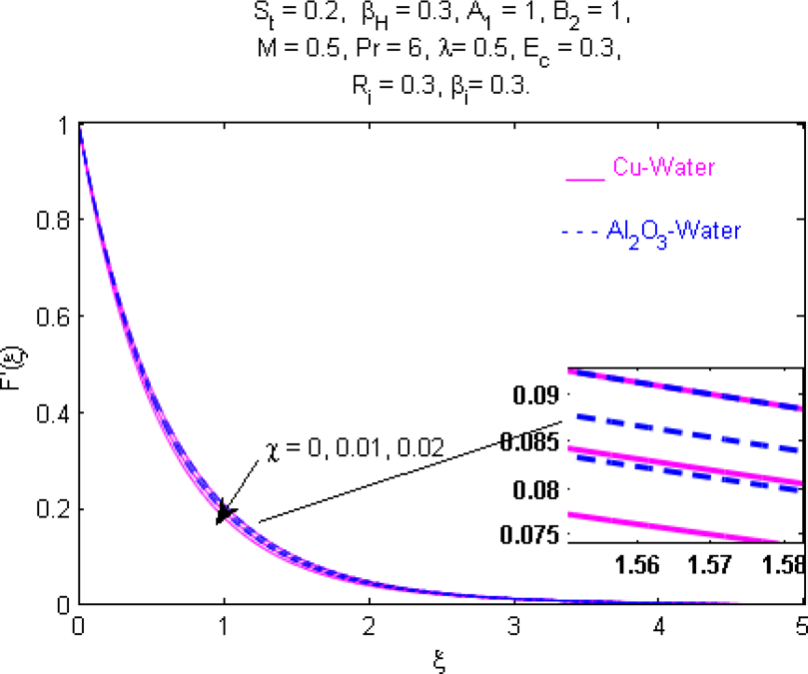
Velocity $F^{\prime } (\xi )$ response for $\chi$.

**Figure 9:**
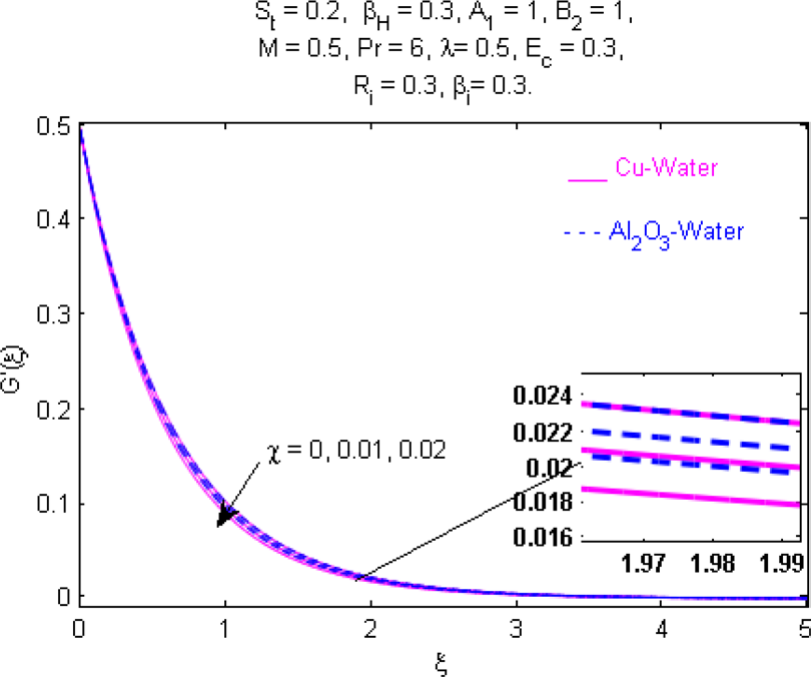
Velocity ${\text{G}}^{\prime } (\xi )$ response for $\chi$.

**Figure 10:**
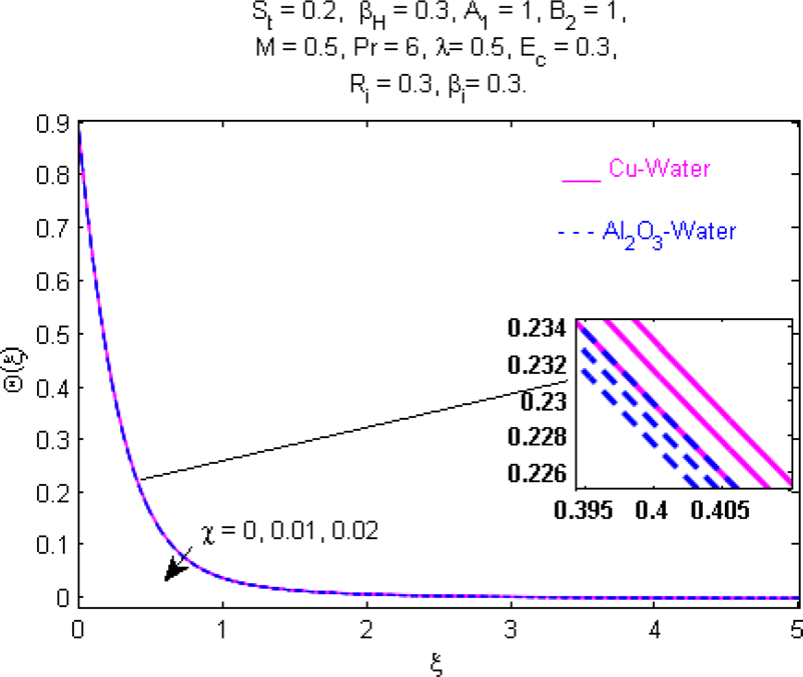
Temperature $\Theta (\xi )$ response for $\chi$.

**Figure 11:**
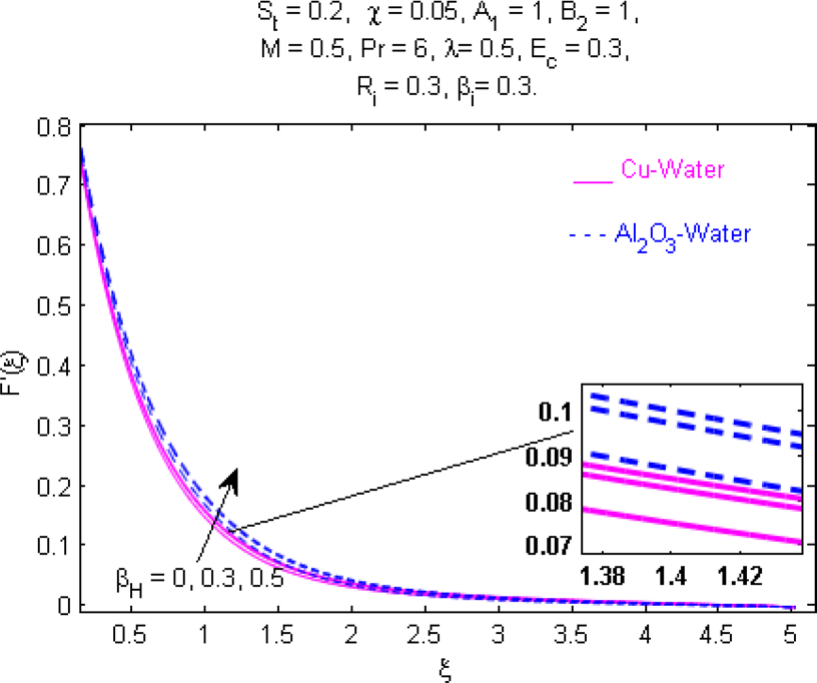
Velocity ${\text{F}}^{\prime } (\xi )$ response for $\beta_{H}$.

**Figure 12:**
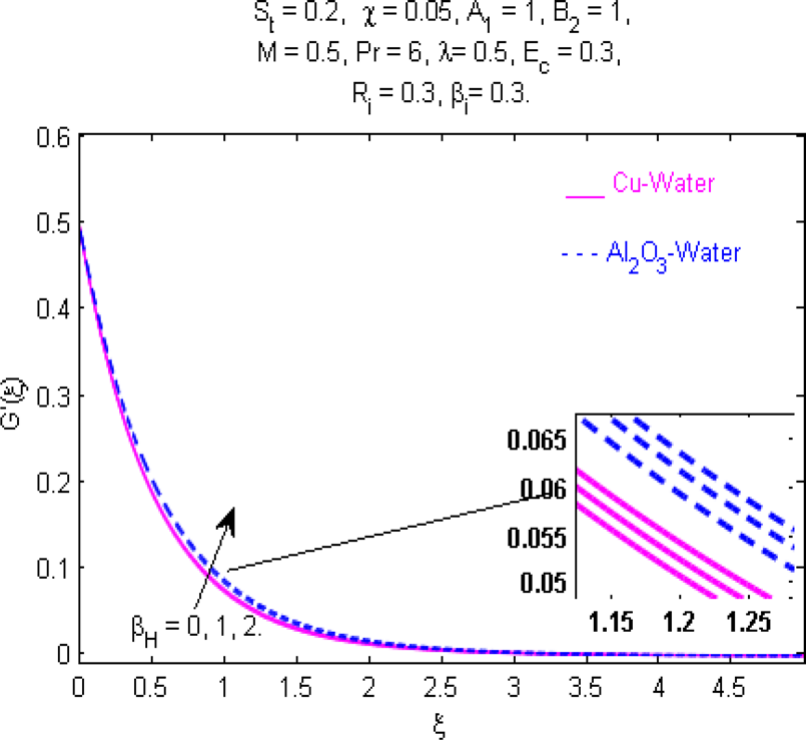
Velocity ${\text{G}}^{\prime } (\xi )$ response for $\beta_{H}$.

**Figure 13:**
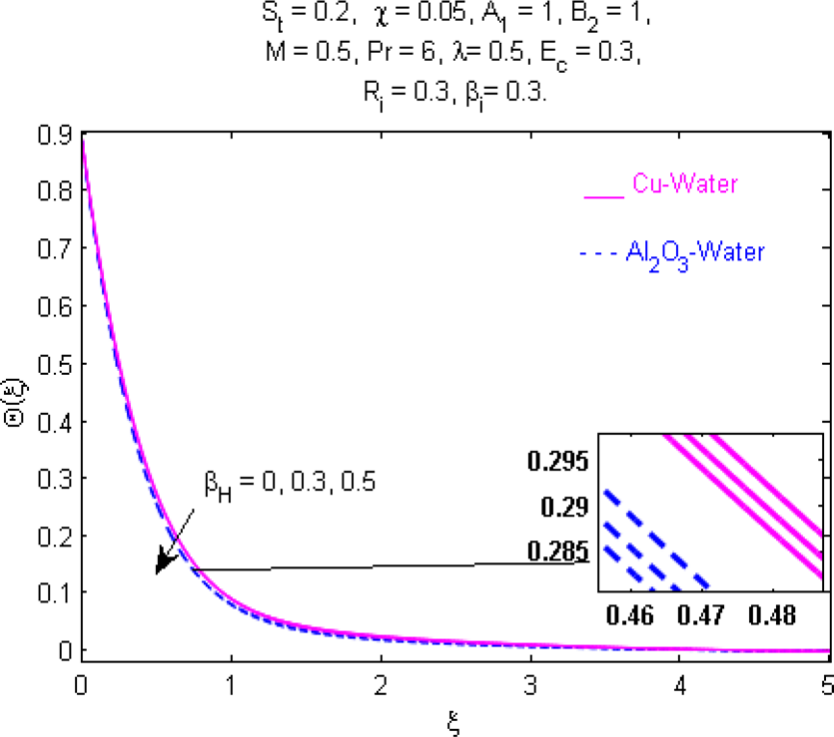
Temperature $\Theta (\xi )$ response for $\beta_{H}$.

**Figure 14:**
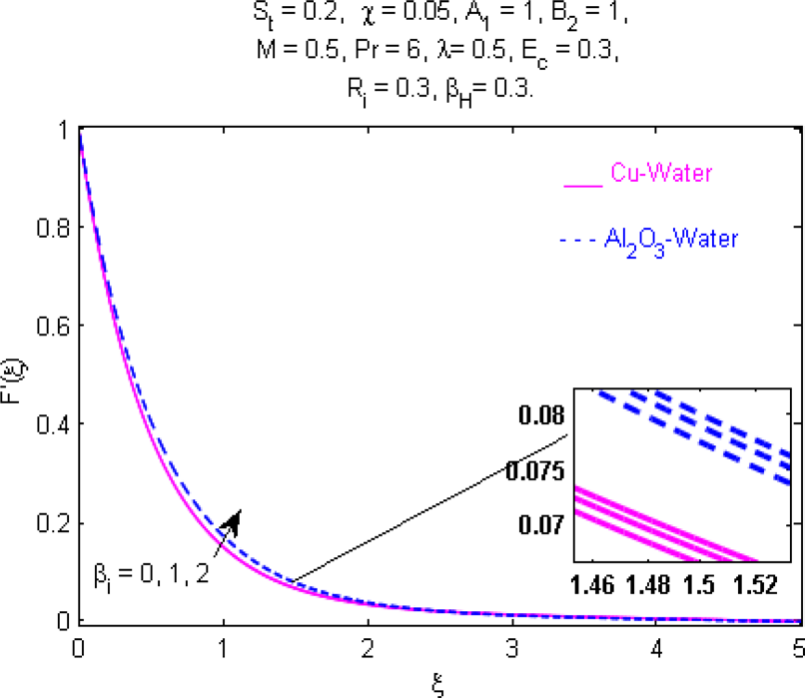
Velocity ${\text{F}}^{\prime } (\xi )$ response for $\beta_{i}$.

**Figure 15:**
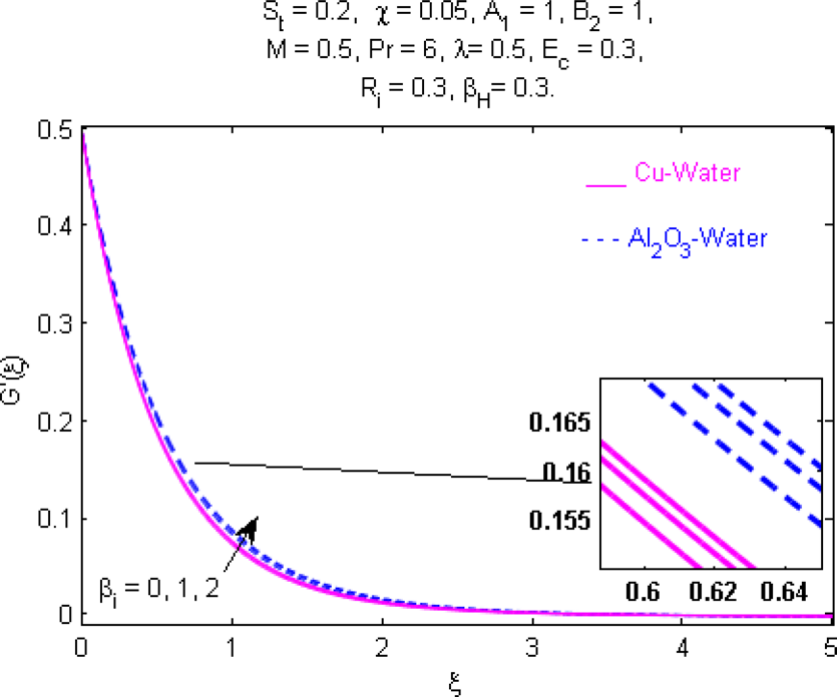
Velocity ${\text{G}}^{\prime } (\xi )$ response for $\beta_{i}$.

**Figure 16:**
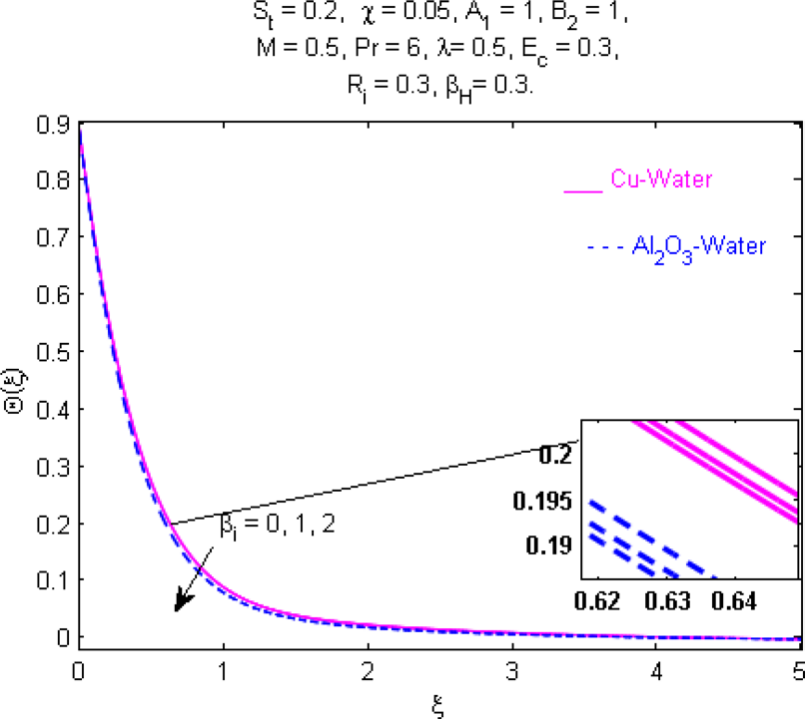
Temperature $\Theta (\xi )$ response for $\beta_{i}$.

**Figure 17:**
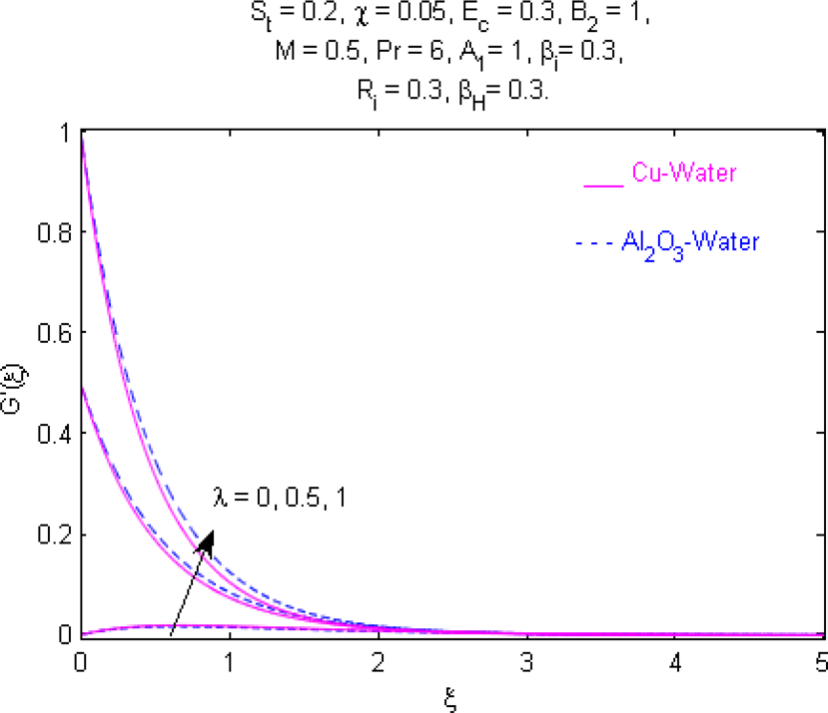
Velocity ${\text{G}}^{\prime } (\xi )$ response for $\lambda$.

**Figure 18:**
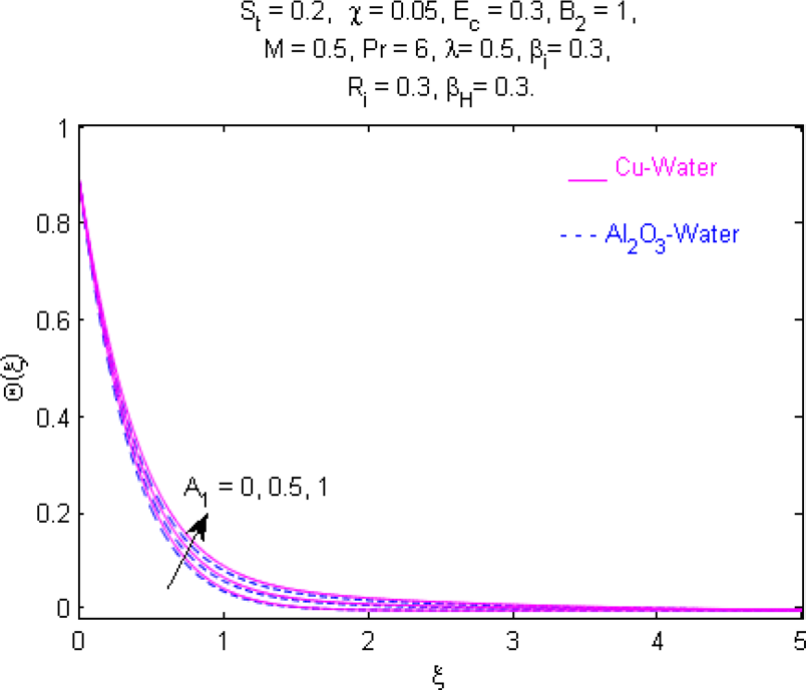
Temperature $\Theta (\xi )$ response for $A_{1}$.

**Figure 19:**
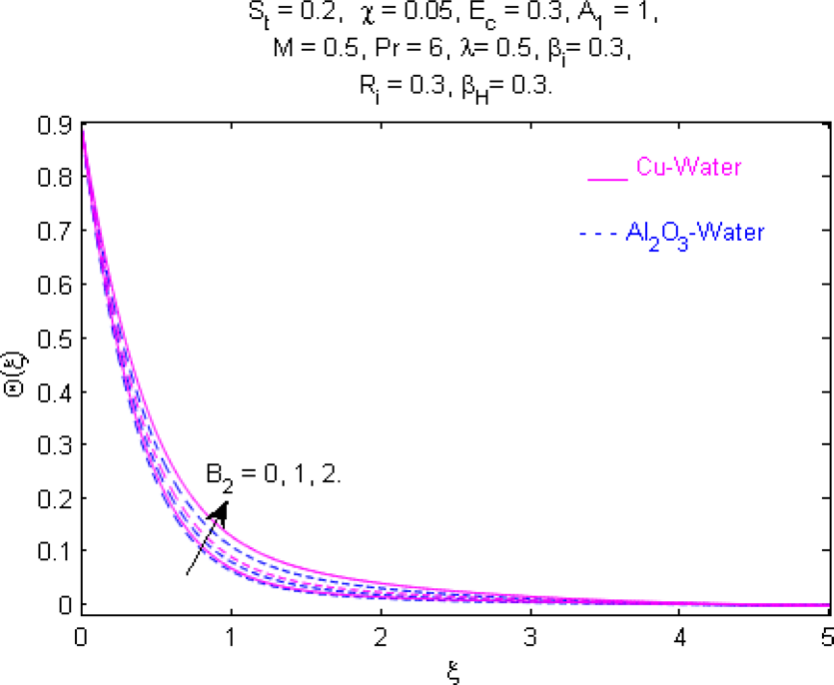
Temperature $\Theta (\xi )$ response for $B_{2}$.

**Figure 20:**
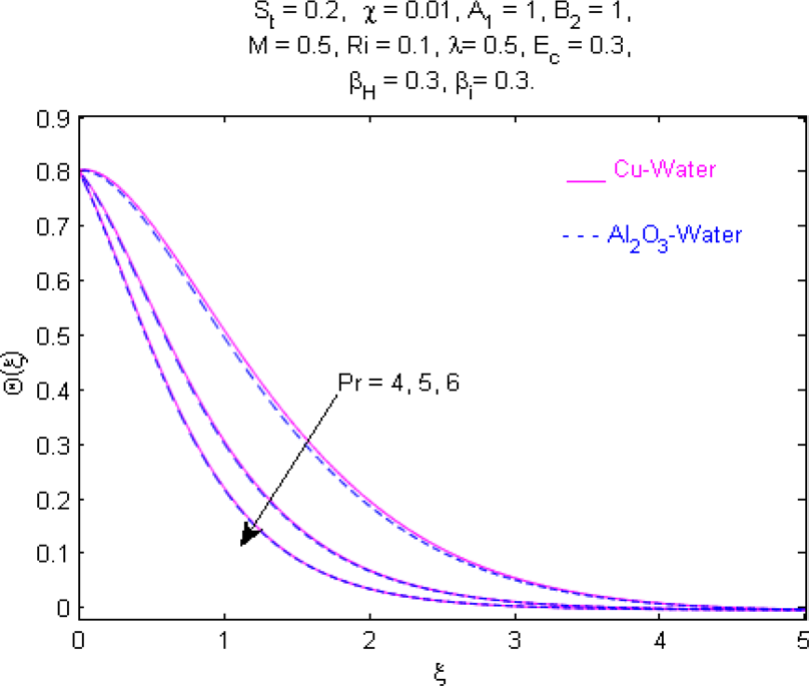
Temperature $\Theta (\xi )$ response for $\Pr$.

**Figure 21:**
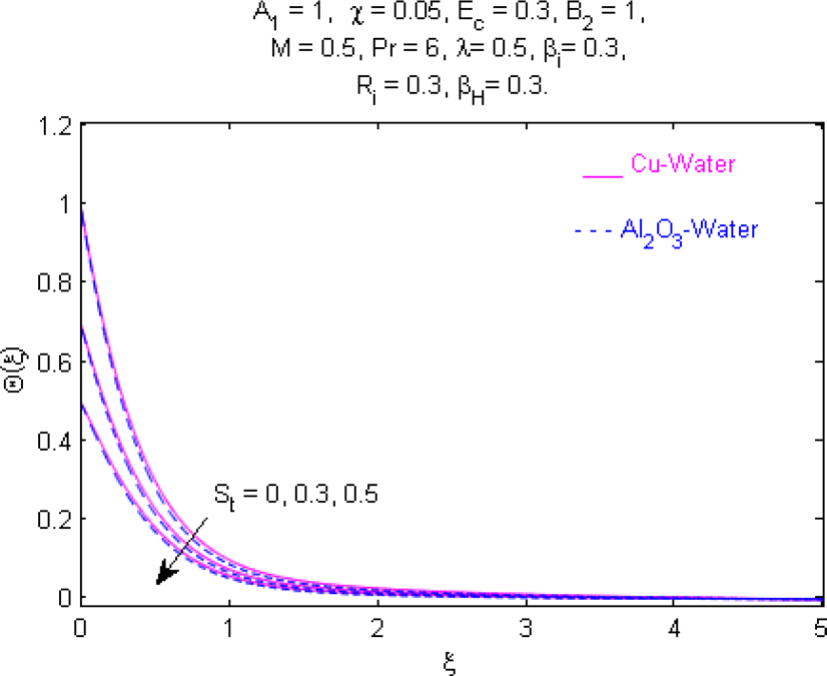
Temperature $\Theta (\xi )$ response for $S_{t}$.

**Figure 22:**
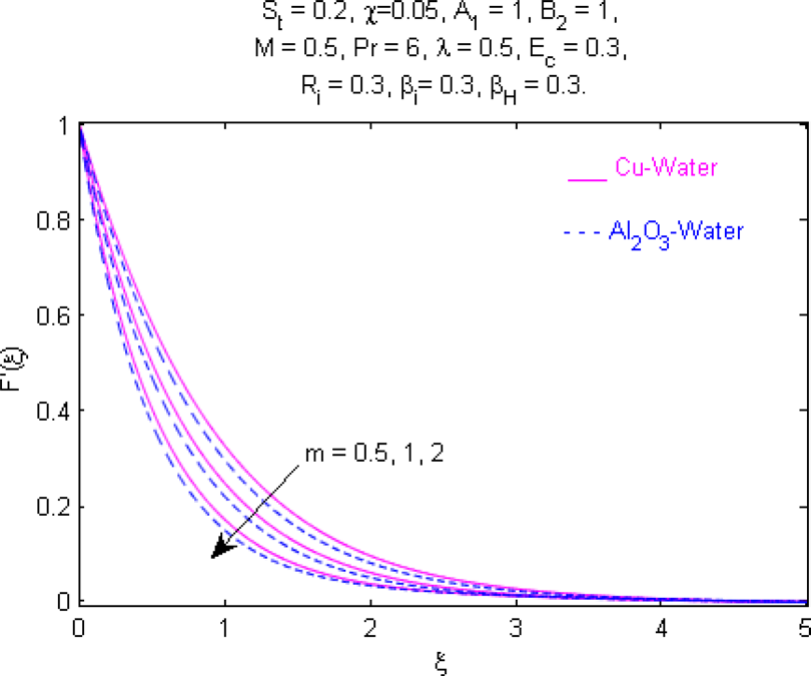
Velocity $F^{\prime } (\xi )$ response for $m$.

**Figure 23:**
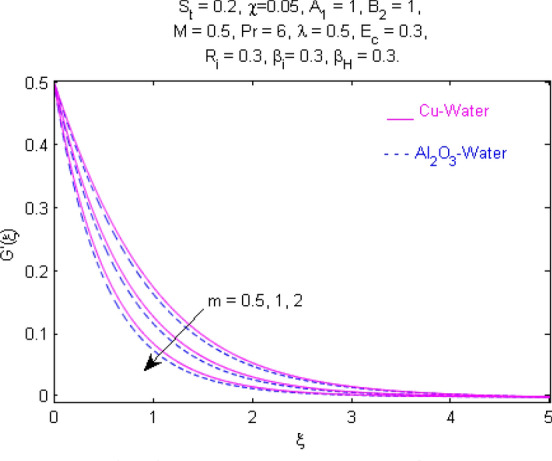
Velocity ${\text{G}}^{\prime}(\xi )$ response for $m$.

**Figure 24:**
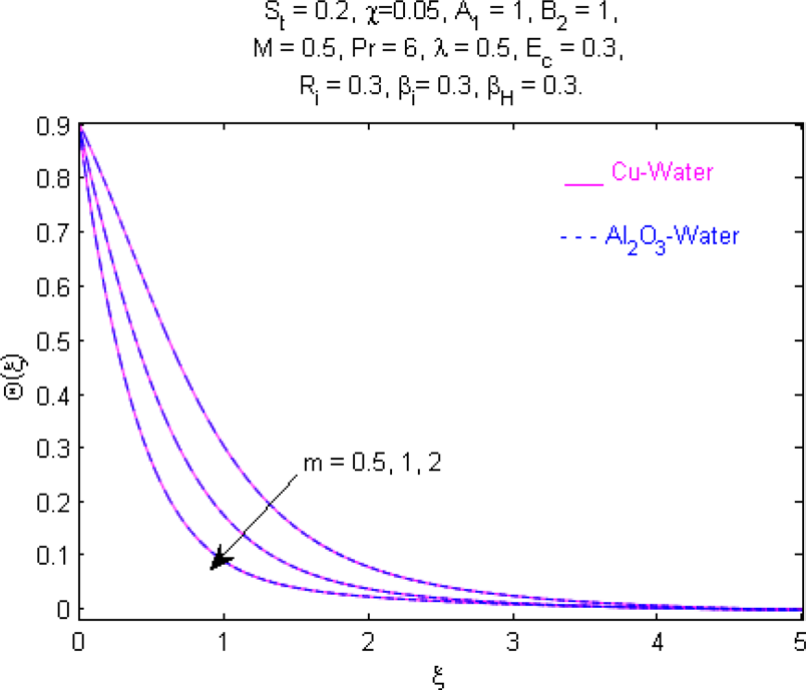
Temperature $\Theta (\xi )$ response for $m$.

**Figure 25:**
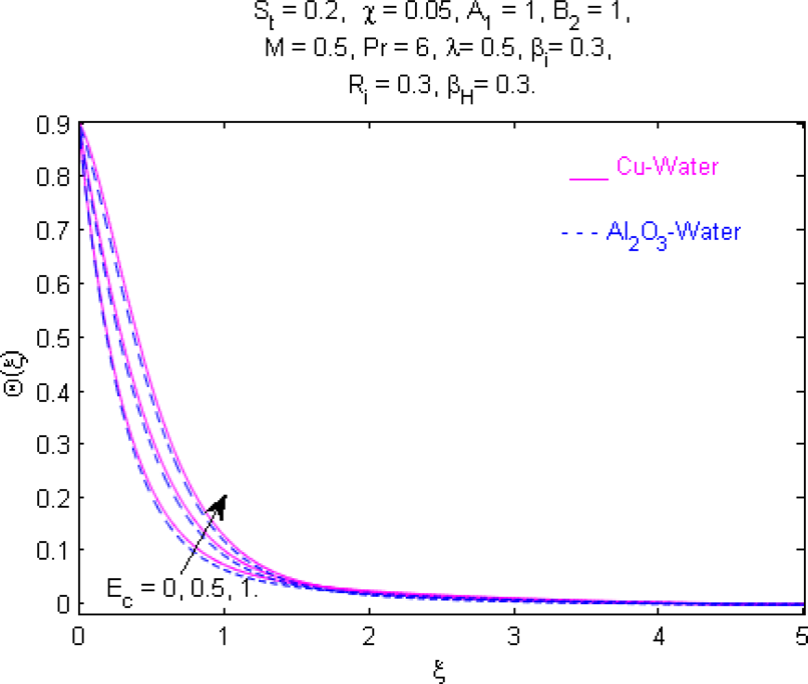
Temperature $\Theta (\xi )$ response for $E_{c}$.

This section (Figs. 2, 3, 4, 5, 6, 7, 8, 9, 10, 11, 12, 13, 14, 15, 16, 17, 18, 19, 20, 21, 22, 23, 24, 25) is devoted to witnessing the behavior of the numerous arising parameters versus involved profiles. The results of the velocity and temperature field are depicted for various parameters through Figs. 2, 3 and 4. The magenta color represents copper and blue signifies aluminum oxide with base fluid water. Figures 2, 3 and 4 demonstrate the impression of the Richardson number on velocity and temperature distributions. Higher estimates of the Richardson number trigger the fluid velocity to upsurge. The buoyancy force plays a pivotal role to attain a favorable pressure gradient. This improved buoyancy force supports the fluid movement in the upright direction that eventually enhances the fluid velocity. But temperature distribution decreases when incrementing in Richardson number. As the gap amid the fluid temperature at the surface and the far away from the surface in decreased gradually. Therefore, it causes the fluid temperature to decline. It is also interesting to observe that the temperature is dominant in nano-sized metal as compared to nano-sized metal oxide with base fluid. Moreover, the velocity of metal oxide nanoparticles is higher as compared to the velocity of metal nanoparticles in nano-fluids. Figures 5 and 6 illustrate the behavior of partially ionized nanofluid flow affected by the variation in magnetic intensity. From these figures, it is inferred that the strong magnetic field lowers the flow in both *x-* and *y-*directions due to the Lorentz force. The impact of the magnetic field strength on the temperature profile is shown in Fig. 7. The fluid temperature upsurges for mounting estimates of the magnetic parameter. The magnetic field intensity has a strong influence on the temperature of nanofluid. There is a directly proportionate relation between the Joule heating phenomenon and the intensity of the magnetic field. The rise in magnetic intensity causes increased heat dissipation and as a result of the Joule heating process, the temperature of nanofluid surges. Figures 8, 9 and 10 are drawn to show the influence of the volume fraction parameter on the velocity and temperature profiles. It is witnessed that both profiles demonstrate a decreasing trend for the volume fraction parameter. The reduction in the collision of ions and electrons because of the higher concentration of solid nanoparticles in a partially ionized liquid is the reason for the decrease in temperature distribution. The association of the Hall parameter with the fluid velocity and temperature is portrayed in Figs. 11, 12 and 13. It is evident in Fig. 11 that if the Hall parameter increases, the movement of magnetized partially ionized liquid in the *x-*direction is enhanced. The product of electron collision time and their respective frequency is known as the Hall parameter. An increase in any one or both of these two boosts the Hall parameter resulting in Hall current known as Hall force. It is experimentally proven that the Hall and magnetic forces are opposite to each other. The fact has also been thematically verified in the current study. Consequently, for an increase in the Hall parameter, an upsurge is expected in the velocity of the fluid in the *x-*direction. Similar behavior in the *y-*direction is seen in Fig. 12. Therefore, the current theoretical results are entirely consistent with the basic laws of physics. The impact of Hall current on the temperature profile is depicted in Fig. 13. It is noticed that fluid temperature is on the decline for growing estimates of Hall current parameter. Figures 14 and 15 portray the influence of the ion slip parameter on *x-* and *y*-components of the velocity of the fluid, respectively. The similar impacts of the Hall and Ion slip parameters on the nanofluid velocities along *x-* and *y*-directions are observed. Figure 16 illustrates the influence of the Ion slip parameter on the temperature of the partially ionized liquid. In Eq. (15), according to Ohm’s Law the Hall and ion slip parameters have an inverse relation with temperature profile. Hence, the dissipation of heat is reduced if Hall and Ion slip parameters increase consequently the temperature of nanofluid is reduced. The impact of the stretching rate parameter on the velocity along *y-*direction is portrayed in Fig. 17. The fluid velocity triggers for large estimates of the stretching rate parameter. It is an accepted truth that once the stretching in enhanced, the velocity of the fluid is escalated. Figures 18 and 19 are outlined to witness the impact of non-uniform heat generation parameter on the temperature field. The temperature of ionized nanofluid is significantly is raised because of non-uniform heat generation. Higher estimates of non-uniform heat generation produce more heat that eventually raises the fluid temperature. The behavior of the temperature profile for varied estimates of the Prandtl number is given in Fig. 20. It is witnessed that an increase in the Prandtl number lowers the fluid temperature. It is quite evident that the rise in the Prandtl number declines the thermal boundary layer thickness that eventually lowers the fluid temperature. Figure 21 exhibits the temperature of nanofluid for growing values of the thermal stratification parameter. A decline in the temperature of the fluid is observed for the thermal stratification parameter. Higher estimates of the thermal stratification will lower the gap between the surface and ambient temperatures. Thus, fluid temperature and the thickness of the thermal boundary layer is reduced for escalating impacts of the stratification parameter. The relationship of the Power law index with the fluid velocities and the temperature is depicted in Figs. 22, 23 and 24. A decline in the velocities profiles and temperature distribution is seen for the Power-law index. Higher estimates of the Power-law index for the stretched surface will affect the fluid velocities in both directions. A similar trend is observed for the temperature profile. Figure 25 illustrates the influence of the Eckert number on the fluid temperature. It is seen that the temperature of the fluid escalates for enhanced values of the Eckert number. As Eckert number is the coefficient of viscous dissipation and Joule heating terms, consequently, enhancement in values of Eckert number corresponds to the rise in heat dissipated owing to friction force and Joule heating process. Hence, the fluid temperature is increased due to the supplemented dissipated heat to the fluid as the outcome of friction and Joule heating.

Table 5 is constructed to witness the behavior of the Hall and ion slip parameters, stratification parameter, non-uniform heat source/sink, and Richardson number on the surface drag coefficients in both directions and on the rate of heat transfer. Due to the rise in the thermal stratification parameter, the wall shear stresses have displayed increment and wall heat flux is reduced. Moreover, wall shear stresses are higher for Copper-based nanofluid than for Alumina based nanofluid, but the opposite behavior is observed for heat flux. As the Richardson number enhances, it lowers the wall shear stresses for nanofluid, and the wall heat flux increase. A decreasing trend of the wall shear stresses and an increasing trend for heat flux for higher estimates of Hall and ion slip parameter is also observed. The impact of non-uniform heat source/sink on the shear stresses and heat flux of the wall is also witnessed. The wall shear stresses, and wall heat flux decrease when the heat source/sink parameter is enhanced. A comparison of the outcomes is depicted in special cases with already published work Khan et al.^22^ is given in Table 6. An excellent concurrence between the two results is attained here.

**Table 5:** Numerical outcomes of Nuesslt number and Skin fraction for Cu-water and Al_2_O_3_-water.

Nanofluid		Cu-water			Al_2_O_3_-water		
		$- ({\text{Re}} )^{ - 0.5} C_{{F_{x} }}$	$- ({\text{Re}} )^{ - 0.5} C_{{G_{y} }}$	$({\text{Re}}_{x} )^{0.5} Nu$	$- ({\text{Re}} )^{ - 0.5} C_{{F_{x} }}$	$- ({\text{Re}} )^{ - 0.5} C_{{G_{y} }}$	$({\text{Re}}_{x} )^{0.5} Nu$
$R_{i}$	0	0.9865580	0.4939604	0.6692664	0.9640008	0.4829222	0.6943682
	0.5	0.9001704	0.3948557	0.7593171	0.8778813	0.3844143	0.7814454
	1	0.8197464	0.3044374	0.8358658	0.7975616	0.29430146	0.8561931
$\beta_{H}$	0	0.9355860	0.4336869	0.7219442	0.91322970	0.4217813	0.7449328
	0.5	0.9331802	0.4328937	0.7253545	0.9107874	0.4222625	0.7484524
	1.5	0.93121469	0.4324361	0.7295306	0.9087326	0.4230885	0.7527689
$\beta_{i}$	0	0.9341973	0.4338658	0.7228813	0.9118499	0.4232733	0.7458963
	0.5	0.9339545	0.4332610	0.7245269	0.9115951	0.4226431	0.7475991
	1	0.9335255	0.4324607	0.7266412	0.9111454	0.4218090	0.7527689
$S_{t}$	0.3	0.9203101	0.4187136	1.2566311	0.8979185	0.4080877	1.2806838
	0.5	0.9340502	0.4334810	0.7239328	0.9116955	0.4228723	0.7469843
	0.7	0.9478330	0.4483019	0.1924017	0.9255152	0.4377101	0.2144482
$A_{1}$	0	0.9356342	0.4354722	0.7627998	0.9133078	0.4248912	0.7860117
	0.3	0.9332574	0.4324850	0.7044834	0.9108885	0.4218625	0.7274547
	0.5	0.9316704	0.4304922	0.6655528	0.9092732	0.4198421	0.6883641
$B_{2}$	0	0.9357673	0.4357486	0.7678840	0.9133781	0.4250859	0.7902936
	0.3	0.9340502	0.4334810	0.7239323	0.9116947	0.4228723	0.7469843
	0.5	0.9327555	0.4317705	0.693078	0.9104283	0.4212042	0.7166186

**Table 6:** Validation of numerical outcome of $$- ({\text{Re}} )^{ - 0.5} C_{{F_{x} }}$$, $$- ({\text{Re}} )^{ - 0.5} C_{{G_{y} }}$$ and $$({\text{Re}} )^{0.5} Nu$$, when $$\beta_{i} = \beta_{H} = 0.3,$$ $$\Pr = 7,$$$$R_{i} = A_{1} = B_{2} = S_{t} = \chi = M = 0.$$

$n$	$\lambda$	Khan et al.^22^			Current results		
		$- ({\text{Re}} )^{ - 0.5} C_{{F_{x} }}$	$- ({\text{Re}} )^{ - 0.5} C_{{G_{y} }}$	$({\text{Re}} )^{0.5} Nu$	$- ({\text{Re}} )^{ - 0.5} C_{{F_{x} }}$	$- ({\text{Re}} )^{ - 0.5} C_{{G_{y} }}$	$({\text{Re}} )^{0.5} Nu$
1	0	1	0	3.072251	1	0	3.071221
	0.5	1.224742	0.612371	3.762724	1.225734	0.612867	3.762342
	1	1.414214	1.414214	4.344779	1.414682	1.414682	4.344585
3	0	1.624356	0	4.968777	1.624744	0	4.968470
	0.5	1.989422	0.994711	6.085485	1.989540	0.994770	6.085350
	1	2.297182	2.297182	7.026913	2.297232	2.297232	7.026710

## Conclusion

In this study, we have investigated the flow of hybrid nanofluid comprising Copper and Aluminum oxide nanoparticles that are immersed into the base fluid water over a nonlinear bi-directional stretched surface. The subject flow is influenced by the Hall and Ion slip impacts amalgamated with the non-uniform source/sink. The thermal stratification condition is taken at the boundary of the surface. The system of transformed differential equations is tackled by the MATLAB software bvp4c function. The salient outcomes of the problem are shown through graphs and Tables. The significant observations of this novel mathematical model are appended below:

- An important role is played by the effective thermal conductivity in the form of increased heat transfer in nanofluid flow. Maximum effective thermal conductivity is seen by Cu nanoparticles. Also, more heat transfer is witnessed for the Cu nanoparticles in comparison to the Al_2_O_3_.
- It is noticed that the velocity field of the nanofluid increases due to an escalation in the Richardson number. The buoyancy force plays a fundamental role to achieve a favorable pressure gradient. This enhanced buoyancy force strengthens the fluid motion in the upright direction that ultimately enriches the fluid velocity. But temperature distribution decreases for incremented Richardson number.
- The temperature of nanofluid is reduced for the enhanced estimates of the stratification parameter.
- An increase in the temperature of nanofluid is observed when the magnetic field intensity is strengthened. This enchantment in the temperature of the liquid is because of an increase in Ohmic and viscous dissipations.
- Due to the rise in the thermal stratification parameter, the wall shear stresses are incremented, and wall heat flux is reduced. Moreover, wall shear stresses are higher for Copper nanofluid than for Alumina nanofluid, but the opposite behavior is shown for heat flux.
